# Identifying the diagnostic utility of artificial intelligence for elbow effusion detection: A systematic review and meta-analysis

**DOI:** 10.1007/s10140-026-02445-7

**Published:** 2026-02-25

**Authors:** Alexis R. Chow, Jonathan Elias, Kunal Shah, Robert Ablove, Sean Mcmillan

**Affiliations:** 1 Rowan-Virtua School of Osteopathic Medicine, Stratford, NJ USA; 2 Futures Forward Research Institute, Toms River, USA NJ; 3 UBMD Orthopaedics and Sports Medicine, Buffalo, NY USA; 4 Virtua Health System, Marlton, NJ USA

**Keywords:** Artifical intelligence, Elbow effusion, Diagnostic radiology

## Abstract

Introduction: Elbow fractures are often difficult to assess on radiographs, and joint effusions may be the only indication of a fracture. As radiologists may not always be available in the acute care setting, the use of artificial intelligence (AI) for effusion detection may be beneficial. This systematic review and meta-analysis analyzes the sensitivity and specificity of AI for elbow effusion detection and compares them to physician radiographic interpretations. We hypothesize there will be no significant differences between the diagnostic utility of AI and physicians. Methods: A systematic review and meta-analysis was performed on five databases (Cochrane, Embase, Scopus, PubMed, Web of Science). Included studies reported numbers of true positives, true negatives, false positives, false negatives, the number of radiographs, sensitivity, specificity, and positive and negative predictive values. A bivariate random-effects meta-analysis was performed in R Studio v.4.5.1 to calculate sensitivity, specificity, and area under the curve (AUC), as well as 95% confidence intervals for sensitivity and specificity. Results: Four retrospective studies were included in the analysis, encompassing 5790 radiographs. (2–5) AI software had a sensitivity of 92.7% (95% CI: 75.3–98.2%), specificity of 97.8% (95% CI: 84.3–99.7%), AUC of 97.8%, and normalized AUC of 95.8%. There was minimal heterogeneity between the AI studies (I^2^ = 39.3%). Physicians, including residents and attendings, had a sensitivity of 94.8% (95% CI: 85.5–98.3%), specificity of 96.8% (95% CI: 84.2–99.4%), AUC of 97.9%, and normalized AUC of 94.6%. There was no significant heterogeneity between the physician studies (I^2^ = 0%). There was no significant difference between AI and physicians in sensitivity (*p* = 0.79) or specificity (*p* = 0.82). Discussion: No significant differences were observed between the radiologists and the AI software, with both groups yielding high specificity and sensitivity, indicating comparable performance.

## Introduction

Elbow effusions carry great utility in the detection of many etiologies, including occult fractures at the elbow joint, septic arthritis, inflammatory arthritis, and hemophilia [1–4]. Notably, the “fat pad” sign, identified on approximately 75% of traumatic elbow injuries, may be the only radiographic finding towards a fracture around the elbow [5]. Timely and accurate detection is essential for initiating appropriate management and preventing additional injuries of the elbow [6]. Missed or delayed diagnosis of elbow fractures can lead to long-term complications such as chronic pain, joint stiffness, and post-traumatic arthritis, particularly in pediatric patients, where growth plate involvement may also affect bone development [7–10]. Unfortunately, detecting subtle signs such as a posterior fat pad can be challenging, particularly for non-specialist physicians in emergency or urgent care settings [1, 5, 11]. Variability in training, image quality, and the subjective nature of interpretation further complicate timely diagnosis.

Recent advances in artificial intelligence (AI) have demonstrated its potential in interpreting radiographs with a level of accuracy comparable to human experts, particularly in detecting fractures of the wrist and hip [12–14]. Two previously published systematic reviews and meta-analyses assessed the utility of AI in the detection of fractures in general, finding AI to be comparable to clinicians in the detection of fractures [14, 15]. These developments suggest a growing role for AI as a diagnostic adjunct, especially in settings with limited radiological resources. Despite this progress, the utility of AI in the detection of elbow effusions, particularly in the context of acute trauma, remains understudied. There have been no comprehensive systematic reviews or meta-analyses evaluating AI’s diagnostic performance in this setting, highlighting a critical gap in the literature.

To help close this gap in knowledge, we conducted a systematic review and meta-analysis to determine and compare the sensitivity and specificity of AI to physicians in the detection of elbow effusions. Determining the reliability of AI in this domain could support its integration into acute care settings, potentially enabling faster diagnosis, improving patient triage, and aiding clinicians where radiology expertise is limited, ultimately yielding superior patient outcomes. We hypothesize that there will be no significant differences between radiologists and AI in the sensitivity and specificity of elbow effusion detection.

## Methods

### Screening

A systematic review and meta-analysis was performed using the Preferred Reporting Items for Systematic Reviews and Meta-Analyses (PRISMA) 2020 guidelines [16]. Five databases (PubMed, Cochrane, Embase, Scopus, and Web of Science) were queried using a Boolean string of keywords and MeSH terms concerning the use of artificial intelligence for the detection of elbow effusions on radiographs. The following Boolean string was used across the five databases: (“artificial intelligence” OR “AI” OR “deep learning” OR “machine learning” OR “neural network” OR “machine intelligence”) AND (“elbow”) AND (“effusion” OR “effusions”) AND (“identification” OR “recognition” OR “detection” OR “diagnosis” OR “diagnose”). Rayyan.ai was used for automated duplicate detection, and 2 independent authors (XX and XX) screened detected duplicates for deletion. Two authors (XX and XX) independently screened the studies based on strict inclusion and exclusion criteria, using a third author (XX) for any ties.

### Inclusion and exclusion criteria

Included studies reported on the ability of AI to diagnose elbow effusions on radiographs. The AI group included neural networks, deep learning algorithms, or any technology that can learn over time. Included studies reported numbers of true positives, true negatives, false positives, and false negatives or reported the appropriate amount of information so that the aforementioned values could be calculated. Studies were excluded if they tested the diagnostic utility of AI to diagnose elbow effusions via other imaging modalities, did not compare to physicians, or lacked sufficient data to deduce the true positives, true negatives, false positives, or false negatives.

### Statistical analysis

A bivariate random-effects meta-analysis was conducted in RStudio v.4.5.1 using the Meta-Analysis of Diagnostic Accuracy (mada) library to calculate the sensitivity, specificity, and area under the curve (AUC) for both AI software and physicians [17]. 95% confidence intervals were calculated and recorded for specificity and sensitivity. Receiver operating characteristic (ROC) curves were generated based on the sensitivities and false positive rates of both groups. I^2^ was calculated following the Zhou and Dendukuri approach to estimate heterogeneity between the studies [18].

For comparison between the AI software and physicians, a meta-regression using the Meta-Analysis Package for R (metafor) was performed for the specificity and sensitivity [18]. A p-value of < 0.05 was considered a statistically significant difference between the two groups. An analysis of accuracy was also performed using the metafor package for R to find the odds ratio [18].

### Risk of bias and quality of evidence

Risk of bias was assessed using Cochrane’s Risk Of Bias In Non-randomised Studies - of Interventions version 2 (ROBINS-I v2) [19]. Traffic light and summary plots were created with a risk of bias visualization tool (robvis) [20]. Quality of evidence was assessed using the grading of recommendations, assessment, development, and evaluation (GRADE) approach [21].

## Results

### Screening

**Fig. 1:**
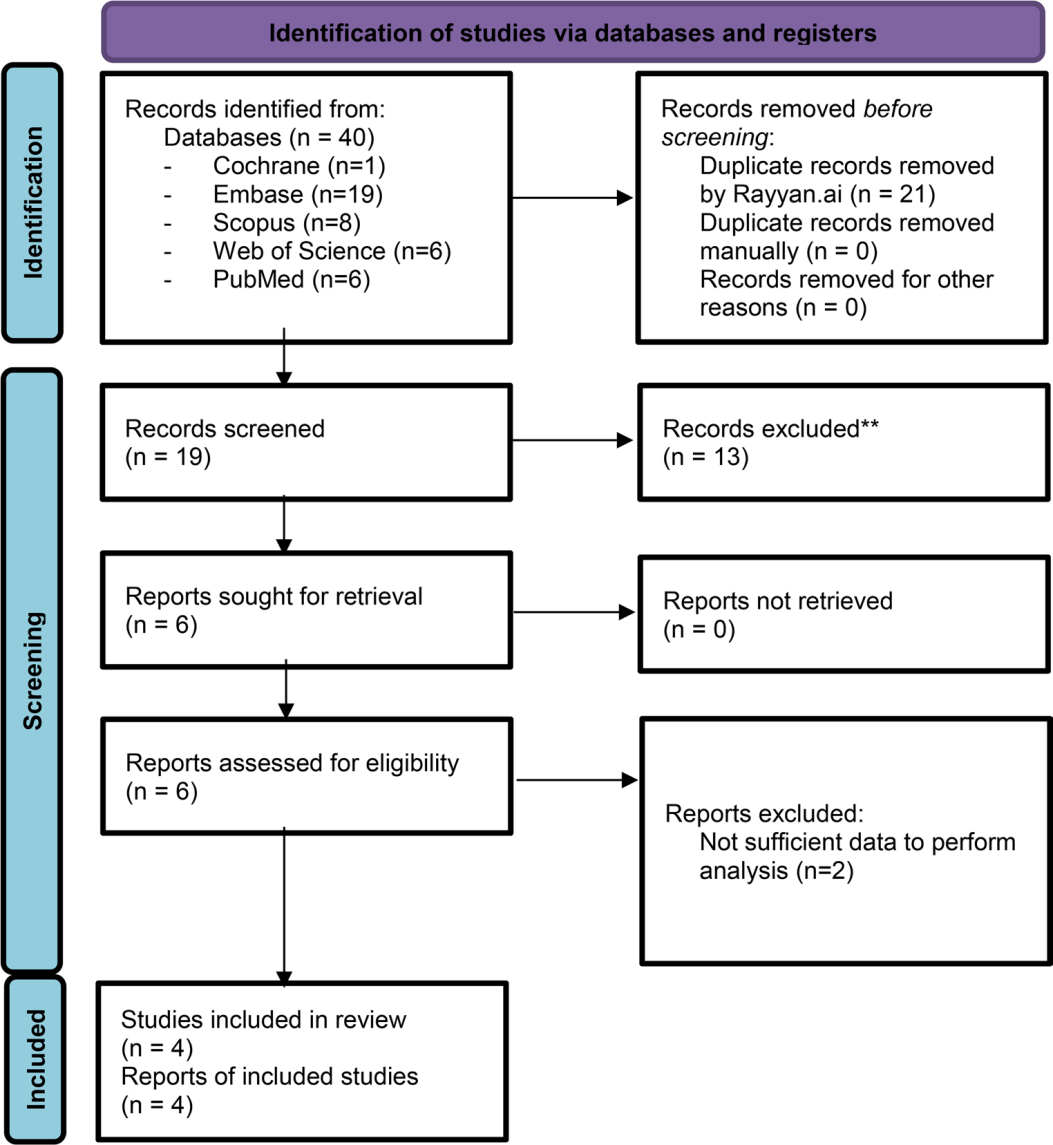
PRISMA Chart. Based on inclusion and exclusion criteria, four studies were included in the final analysis

Four studies were included after screening via strict inclusion and exclusion criteria [22–25]. Some studies may be relevant to the study but did not fit these criteria. Altmann-Schneider et al. 2024 investigated the use of AI for appendicular fracture detection and reported sensitivity and specificity of elbow effusion detection, but did not report the number of elbow radiographs examined and were thus excluded from the study [26]. Dupuis et al. 2024 investigated the use of AI for elbow fracture detection, but only included the true positives of elbow effusion, making it impossible to calculate the other values necessary to run the analysis [27]. The screening process is outlined by the PRISMA chart (Fig. 1).

A summary of the included studies is included in the Summary of Findings (Table 1). The four studies were retrospective studies that compared AI software to different levels of physicians, including residents and attendings [22–25].

**Table 1:** Sumngs tablemary of findi

Study	Title	Study Type	AI Used	Physician Experience	#of radiographs		Age	Male/Female	Outcomes Measured
Diaz-Moreno et al. 2025	Diagnostic Performance of an Artificial Intelligence Software for the Evaluation of Bone X-Ray Examinations Referred from the Emergency Department	Retrospective	Arterys Chest MICA v29.4.0	PGY1 Radiology Resident	28		Not listed separately	Not listed separately	TP, NPV, PPV, sensitivity, specificity, AUC
Huhtanen et al. 2022	Deep learning accurately classifies elbow joint effusions in adult and pediatric radiographs	Retrospective	Deep Convolutional Neural Network	Radiologist	859		33 yr	91/122	TP, FN, FP, sensitivity, NPV, PPV, F1 score
Regnard et al. 2022	Assessment of performances of a deep learning algorithm for the detection of limbs and pelvic fractures, dislocations, focal bone lesions, and elbow effusions on trauma X-rays	Retrospective	BoneView TM	Radiologist	4774		30% pediatrics	2435/2339	TP, TN, FP, FN, sensitivity, specificity, AUC, F1 score, precision, accuracy
England et al. 2019	Detection of Traumatic Pediatric Elbow Joint Effusion Using a Deep Neural Network	Retrospective	Deep Convolutional Neural Network	PGY5 Radiology Resident	129		11.4 year (SD = 5.4)	90/39	TP, TN, FP, FN, sensitivity, specificity

### Diagnostic predictive values

**Fig. 2:**
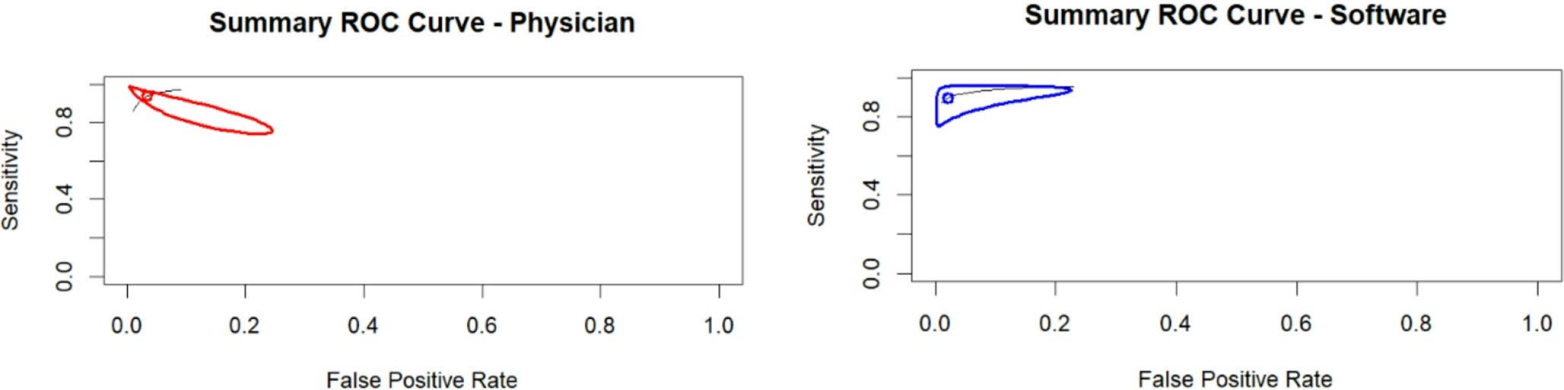
Summary ROC Curves for Physicians and AI Software. Based on the ROC curves, both physicians and AI software have good sensitivity and specificity as both curves predominate in the upper left corner

**Fig. 3:**
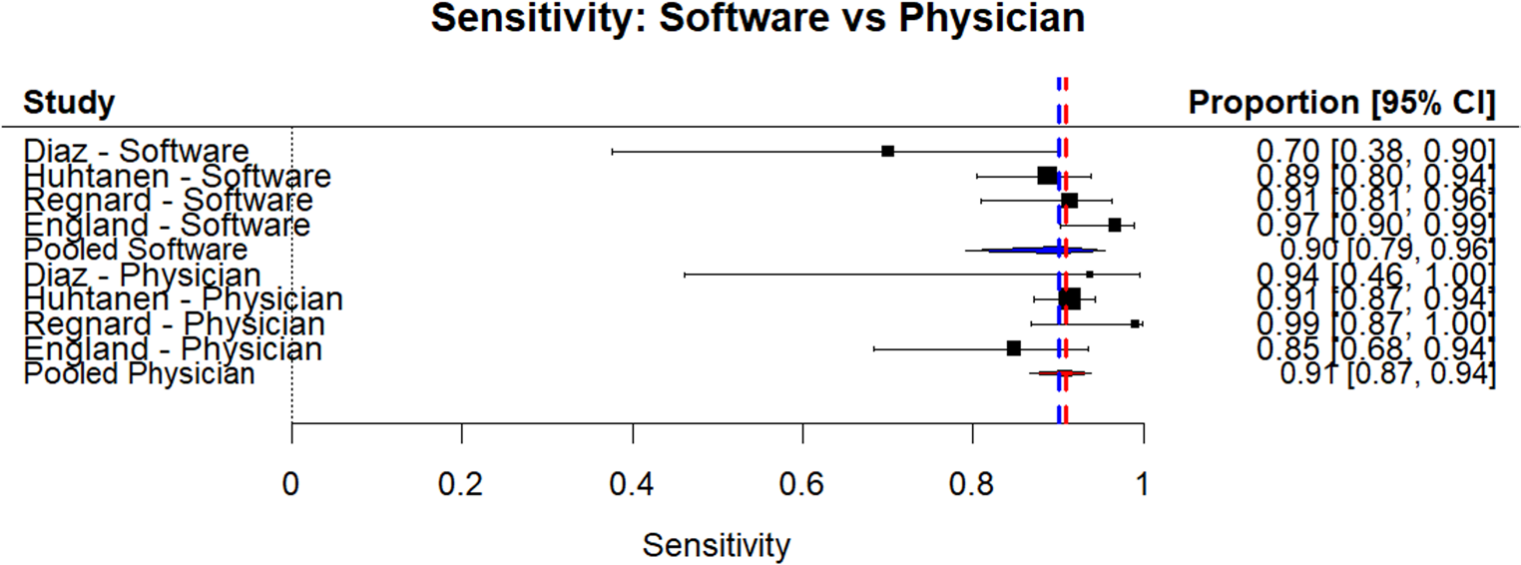
Forest plot of included studies for sensitivity of the software compared to the physicians. The data for AI software is at the top in blue and the data for physicians is at the bottom in red

**Fig. 4:**
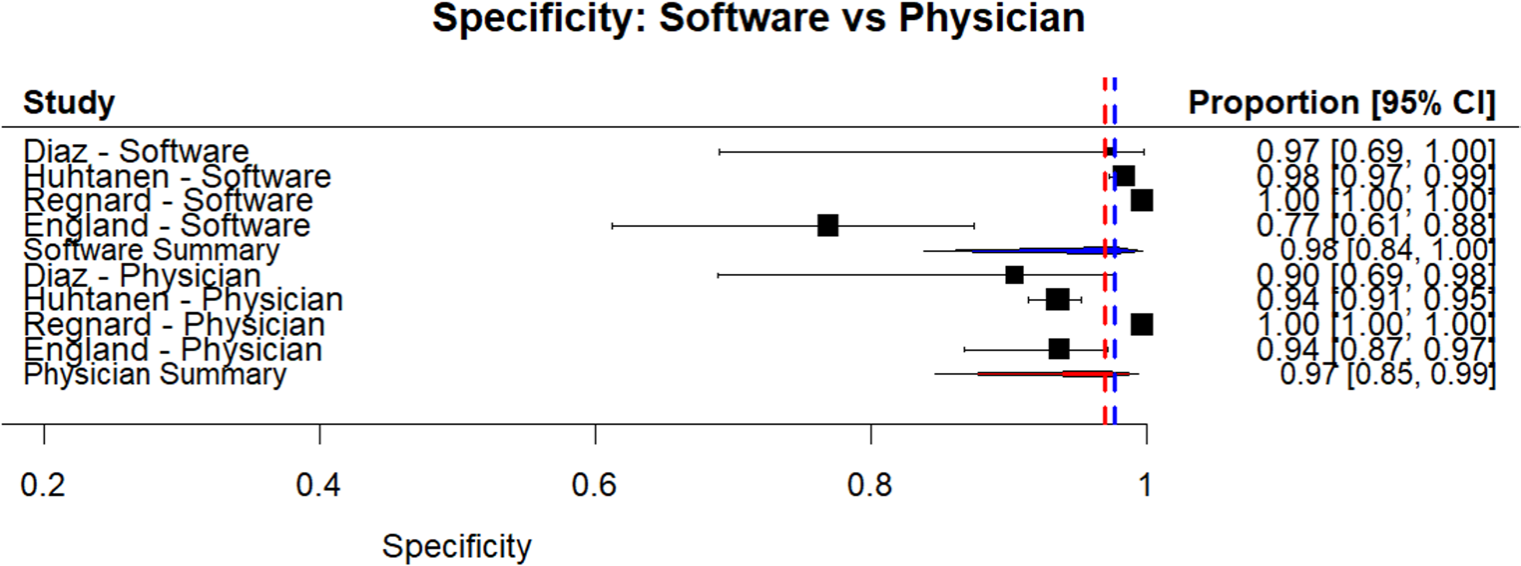
Forest plot of included studies for specificity of the software compared to the physicians. The data for AI software is at the top in blue and data for physicians is at the bottom in red

AI software had a sensitivity of 92.7% (75.3–98.2%), specificity of 97.8% (84.3–99.7%), AUC of 97.8%, and normalized AUC of 95.8%. The ROC curve is depicted in Fig. 2. There was minimal heterogeneity between the sensitivities and specificities of the AI software (I^2^ = 39.3%). Physicians, including residents and attendings, had a sensitivity of 94.8% (85.5–98.3%), specificity of 96.8% (84.2–99.4%), AUC of 97.9%, and normalized AUC of 94.6%. This is depicted in the red ROC curve in Fig. 2. There was no significant heterogeneity between the physician studies (I^2^ = 0%). There was no significant difference between AI and physicians in sensitivity (*p* = 0.79) or specificity (*p* = 0.82) as shown in Figs. 3 and 4. A summary of these findings is included in Table 2.

**Table 2:** Summary of diagnostic predictive values table

Sensitivity Mean (95% CI)	Physicians	AI Software	*P* value
	94.8% (85.5–98.3%)	92.7% (75.3–98.2%)	0.79
Specificity Mean (95% CI)	96.8% (84.2–99.4%)	97.8% (84.3–99.7%)	0.82
AUC	97.9%	97.8%	-
pAUC*	94.6%	95.8%	-
Accuracy	94.3%	99.0%	0.87

*Normalized based on ranges present in included studies

## Accuracy

**Fig. 5:**
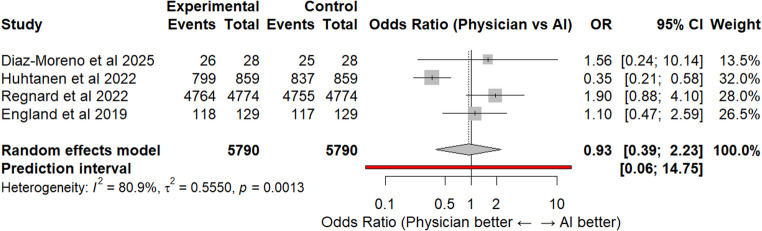
Accuracy of elbow effusion detection

**Fig. 6:**
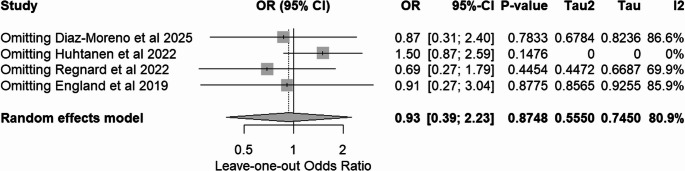
Sensitivity analysis of accuracy of elbow effusion detection

Based on direct pooled analysis, AI showed a higher accuracy of 99.0% compared to 94.3% in the physician group. However, upon further analysis, there was no significant difference in accuracy between physicians and AI, with a p-value of 0.87 and an odds ratio of 0.93 (Fig. 5). There was high heterogeneity between the studies (I^2^ = 80.9%) that could not be attributed to random chance alone. Due to this high heterogeneity, a leave-one-out sensitivity analysis was performed, which indicated that Huhtanen et al., 2022 may be skewing the data, resulting in the odds ratio being more in favor of the AI software (Fig. 6). However, all leave-one-out analyses indicated no significant differences between the physicians and AI software, with the lowest p-value of 0.15.

### GRADE and risk of bias

**Fig. 7:**
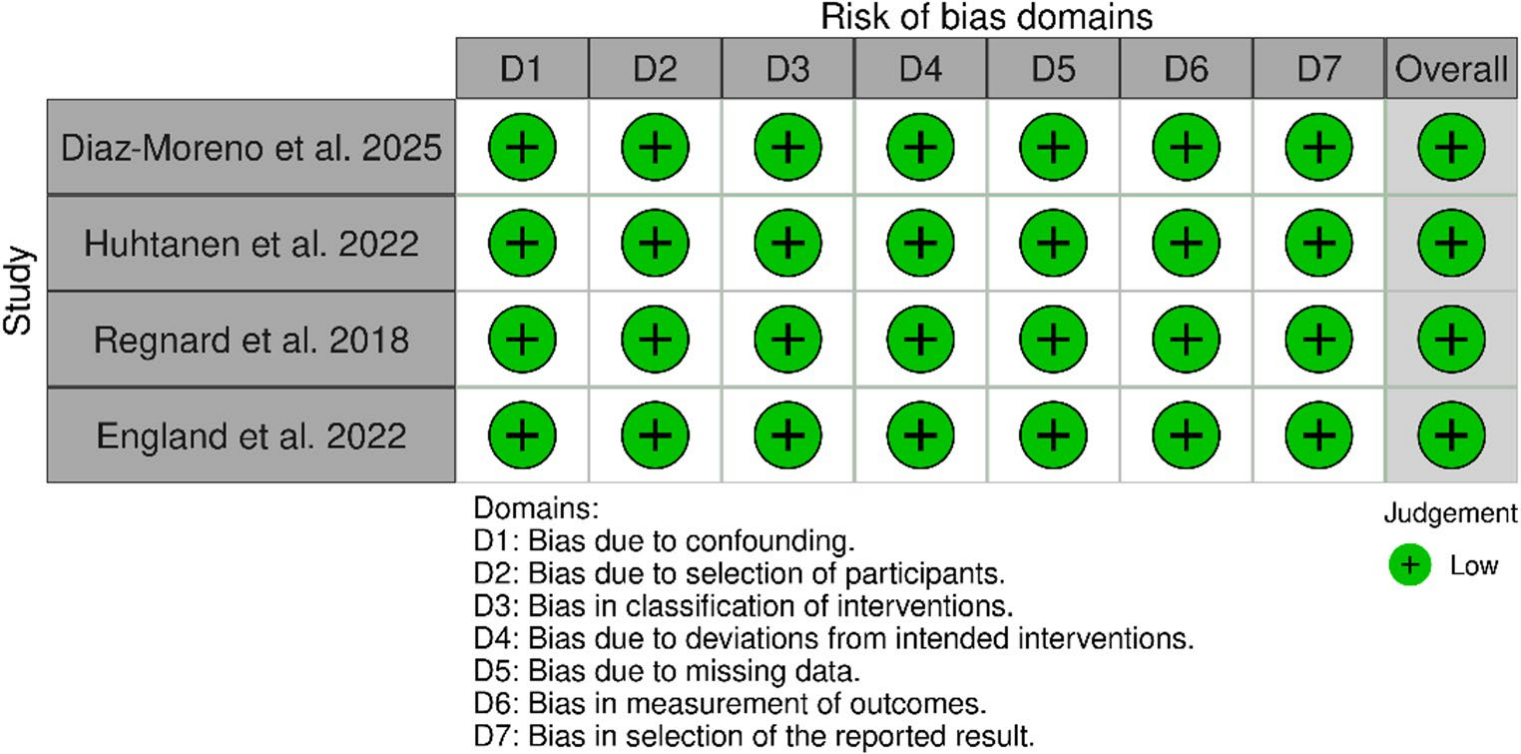
Traffic plot for ROBINS-I v2 of included studies

**Fig. 8:**
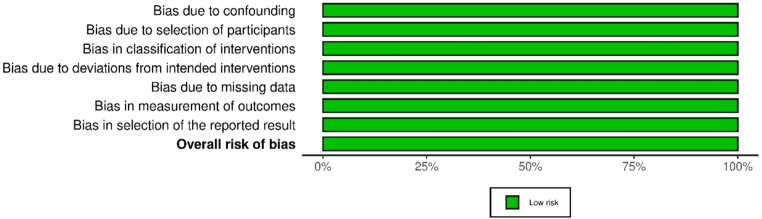
Summary plot of ROBINS-I v2 criteria for included studies

Despite all being retrospective studies, the included studies had a low risk of bias based on Cochrane’s ROBINS-I v2 [19]. Due to the nature of the studies, the physicians and AI software were able to interpret the same X-ray images, making randomization not necessary. Based on the ROBINS-I v2 tool, there was low risk of bias in all studies (Figs. 7 and 8) [19]. There was also minimal heterogeneity between the studies based on the I^2^ values for the main outcomes, sensitivity and specificity, meaning that the studies were generally precise. There was significant heterogeneity in the accuracy of elbow effusion detection, which is most likely due to the different results in Huhtanen et al., 2022, which skewed the data in favor of the AI software. Of note, the definition of ground truth was different between the studies with Diaz-Moreno et al. 2025 and England et al. 2019 indicating the read of the senior radiologist as the ground truth and Huhtanen et al. 2022 and Regnard et al. 2018 using the read of two senior radiologists and a third for tiebreakers as the ground truth, increasing inconsistency between studies [22–25]. The single radiologist read leaves more room for error and thus may result in a less accurate ground truth. In addition, the varying physician experience levels on reading radiographs provided more inconsistency between the studies. The quality of evidence of all of the studies was determined to be moderate based on these factors (Table 3).

**Table 3:** GRADE analysis of included studies

Modified Grading of Recommendations Assessment, Development and Evaluation Criteria for Included Articles								
Author (Year)	Study Design	Risk of Bias	Inconsistency	Indirectness	Imprecision	Publication Bias	Other Factors	Final Grade
Diaz-Moreno et al. (2025)	Retrospective	Low	Serious	Not Serious	Not Serious	Not Serious	N/A	Moderate
Huhtanen et al. (2022)	Retrospective	Low	Serious	Not Serious	Not Serious	Not Serious	N/A	Moderate
Regnard et al. (2022)	Retrospective	Low	Serious	Not Serious	Not Serious	Not Serious	Very high sample size	Moderate
England et al. (2019)	Retrospective	Low	Serious	Not Serious	Not Serious	Not Serious	N/A	Moderate

## Discussion

AI is on the rise within the healthcare sector, with the ever-increasing demand for healthcare efficiency. One promising area for improving efficiency is as a checkpoint for diagnosis. FracNet, RegNetX-3.2GF, RetinaNet, and DenseNet-201 have shown the ability to effectively diagnose a variety of imaging findings comparable to various expert physicians in the field [28–31].

This systematic review and meta-analysis aims to provide a concise understanding of the use of AI for identifying elbow fractures via joint effusion detection. Four studies were included in the bivariate random-effects meta-analysis and meta-regression and odds ratio analysis of accuracy. Physicians from different levels of experience, from first year residents to senior radiologists, were pooled. The primary outcomes were sensitivity, specificity, and AUC, with accuracy as a secondary outcome.

With the sensitivity analysis, physicians performed slightly better by ~ 2% (Physician: 94.8%; AI: 92.7%), meaning that physicians correctly identified more positive cases than AI. Whereas with specificity analysis, AI correctly identified a higher number of actual negative cases (i.e., no fracture) compared to expert physicians (AI: 97.8%; Physicians: 96.8%). Despite the small number of studies included and the sources of heterogeneity within the study, these results suggest that AI may be comparable to certain physicians in detecting elbow effusions and may be used as a tool to improve diagnostic accuracy, though more studies are needed to assess the sensitivity and specificity of combined efforts between physicians and AI.

Global analysis of the diagnostic measure was performed using the area under the curve method. AUC close to 100% means excellent diagnostic performance with AI at 97.8% and physicians at 97.9% meaning both groups performed equally well. When AUC was standardized across the four comparative studies, pAUC for AI was 95.8% whereas for physicians pAUC was 94.6%, indicating both groups had excellent diagnostic accuracy. A heterogeneity analysis yielded a moderate variation among the AI group, whereas none among the physician experts. This was likely due to the use of different AI software, and the results of this study should not be generalized to all AI software.

Accuracy was assessed as a secondary outcome to compile the data of true positives and true negatives. Based on pooled analysis, AI showed a higher accuracy of 99.0% compared to 94.3% in the physician group; however, this may be due to the higher accuracy reported in Regnard et al. 2022, which included a significantly higher number of X-rays than the other studies [25]. The odds ratio for accuracy was OR = 0.96, with physicians having a slightly higher odds of being accurate, though not statistically significant (*p* = 0.87). There was also high heterogeneity within the studies for accuracy, possibly due to the differences in the AI software used, and how the software was validated and tested. Due to the high heterogeneity between studies and the increased possibility for bias, less confidence should be placed on the accuracy results compared to the results for sensitivity, specificity, and AUC.

Historical studies have shown that AI can play a supporting role for physicians in identifying any missed positive or negative identifications. Gasmi et al. 2023 performed a retrospective analysis comparing the diagnostic accuracy of AI to emergency physicians, junior radiologists, and senior radiologists for appendicular fractures among the pediatric population, with AI performing equally to pediatric radiologists and senior residents and better than emergency physicians and junior residents within the field (Sensitivity = 95.6%, specificity = 91.64%) [32]. Duron et al. conducted an appendicular fracture AI study in diagnostic imaging and found that AI demonstrated a gain in sensitivity when used in conjunction with a physician radiologist by 8.7% and an increase in specificity by 4.1% while improving PPV and NPV, that improved as AI was tested and validated, ending with an AOC at 94% [33]. Similarly, Anderson et al. 2024 showed an improved accuracy in identification with AI used in conjunction with a physician [34]. A study by Herpe et al. 2024 shows that with the use of AI, discrepancies within imaging findings were reduced by 17% (*p* < 0.05). While studies are still objectifying these outcomes, with AI use being no different, if not slightly inferior, to physician experts, improvements in such AI systems could be a major revolution in the delivery of patient care [14]. The comparative analysis in this SRMA led to no statistically significant differences between the two groups for sensitivity and specificity (p-value of 0.79 and *p* = 0.82, respectively) for detecting elbow fractures. Further advancement in AI technologies and further studies on the combined effect of AI and trained physicians, based on this and previous studies, may improve diagnostic accuracy and thus allow for timely care of elbow effusion and fractures.

### Limitations

Our study is not without limitations, as the majority of the included studies compare AI accuracy with varying levels of physician experience, ranging from first-year residents to senior radiologists. This variation among the control group affects the certainty of evidence that comes through within the statistical comparison. Though there was not a significant effect on the heterogeneity of the control group through the outcomes studied, pooling the results of physicians at varying levels of experience could skew the data more towards AI if senior radiologists are available and away from AI if junior residents are typically reading X-rays in the emergency setting. There were also different definitions of ground truth, with some studies stating ground truth as the senior radiologist who read the original report, particularly for the studies that studied residents compared to the AI, and the others cited ground truth as defined by two senior radiologists with a third used as a tiebreaker. The single radiologist’s read may be less accurate and thus has potential to skew towards or against AI. Another limitation that arises is the number of images used to compare the two groups. While two studies (Huhtanen et al., 2022 & Regnard et al., 2022) used over 500 images to perform the training and analysis of the AI software, the remaining two studies, Diaz-Moreno et al., 2025 and England et al., 2019, used only 28 and 129 images, respectively, which could have impacted the analysis [22–25]. In addition, all the studies were retrospective in nature, which could introduce additional biases. Due to the measurable heterogeneity and wide confidence intervals, as well as the small number of studies included in this review, it is uncertain whether the ability of AI to distinguish elbow effusions from radiographs is equivocal to that of physicians, and more analyses with larger data sets that distinguish the use of different AI models should be performed.

## Conclusion

This systematic review and meta-analysis indicated that AI software may be comparable to certain physicians on accuracy or specificity for detection of elbow effusions, but AI had non-significantly lower sensitivity.

## Data Availability

Data is available upon request via email to the corresponding author.
